# Hearing Loss and Hair Cell Death in Mice Given the Cholesterol-Chelating Agent Hydroxypropyl-β-Cyclodextrin

**DOI:** 10.1371/journal.pone.0053280

**Published:** 2012-12-28

**Authors:** Mark A. Crumling, Liqian Liu, Paul V. Thomas, Jennifer Benson, Ariane Kanicki, Lisa Kabara, Karin Hälsey, David Dolan, R. Keith Duncan

**Affiliations:** Kresge Hearing Research Institute, Department of Otolaryngology - Head and Neck Surgery, University of Michigan, Ann Arbor, Michigan, United States of America; Dalhousie University, Canada

## Abstract

Cyclodextrins are sugar compounds that are increasingly finding medicinal uses due to their ability to complex with hydrophobic molecules. One cyclodextrin in particular, 2-hydroxypropyl-β-cyclodextrin (HPβCD), is used as a carrier to solubilize lipophilic drugs and is itself being considered as a therapeutic agent for treatment of Niemann-Pick Type C disease, due to its ability to mobilize cholesterol. Results from toxicological studies suggest that HPβCD is generally safe, but a recent study has found that it causes hearing loss in cats. Whether the hearing loss occurred via death of cochlear hair cells, rendering it permanent, was unexplored. In the present study, we examined peripheral auditory function and cochlear histology in mice after subcutaneous injection of HPβCD to test for hearing loss and correlate any observed auditory deficits with histological findings. On average, auditory brainstem response thresholds were elevated at 4, 16, and 32 kHz in mice one week after treatment with 8,000 mg/kg. In severely affected mice all outer hair cells were missing in the basal half of the cochlea. In many cases, surviving hair cells in the cochlear apex exhibited abnormal punctate distribution of the motor protein prestin, suggesting long term changes to membrane composition and integrity. Mice given a lower dose of 4,000 mg/kg exhibited hearing loss only after repeated doses, but these threshold shifts were temporary. Therefore, cyclodextrin-induced hearing loss was complex, involving cell death and other more subtle influences on cochlear physiology.

## Introduction

Cholesterol is a key regulator of cell function, acting as a biochemical substrate and a major component of cell membranes. In the membrane, cholesterol governs bilayer rigidity and fluidity, thereby influencing the ability of membrane components to associate with each other [1]. Cholesterol plays an important role in cell signaling by directly modulating protein function and by facilitating the assembly of molecular complexes into discrete microdomains [2]. Consequently, cholesterol dysregulation causes serious health problems beyond those associated with serum cholesterol levels.

Organs protected by a blood barrier, such as the brain and cochlea, can be severely affected by cholesterol problems despite isolation from serum. Sterol disorders such as Smith-Lemli-Opitz syndrome [3] and Niemann-Pick Type C disease [4] display devastating central neurological phenotypes. These conditions also include sensorineural (peripheral) hearing loss as a part of the spectrum. The brain and cochlea may rely on similar mechanisms to synthesize and regulate cholesterol locally, rendering them both exceptionally poor at meeting the cell-biological demands for cholesterol when these mechanisms go awry.

Little is known about the relationship between membrane cholesterol and cell physiology in the ear. However, several *in vitro* studies have uncovered cholesterol sensitivity in a variety of membrane proteins important to the physiology and health of sensory hair cells in the cochlea. These studies have largely relied on the *in vitro* manipulation of cholesterol using the cyclic oligosaccharide methyl-β-cyclodextrin, whether applied to hair cell preparations or HEK293 cells heterologously expressing hair cell-related proteins. For example, such studies have linked the maturation of hair cell excitability to developmental changes in hair cell cholesterol content [5]. Moreover, in mature hair cells, voltage-gated calcium current is increased while outward potassium current is decreased by cholesterol depletion with methyl-β-cyclodextrin [6]. Cholesterol regulation of membrane proteins is not limited to ion channels. In outer hair cells (OHCs), cholesterol modulates the response properties of prestin, the motor protein that is the molecular basis of the mammalian cochlear amplifier [7]. Taken together, it is apparent that membrane cholesterol is intimately tied to the function of the peripheral auditory system.

Cyclodextrins have been employed widely to manipulate the level of cholesterol for investigating its role in membrane protein function, but these compounds are finding medicinal uses as well. Recently, 2-hydroxypropyl-β-cyclodextrin (HPβCD) has emerged as a candidate therapeutic agent for the lysosomal storage disorder Niemann-Pick Type C disease (NPC), where HPβCD aids in the transport of accumulated cholesterol out of the lysosome [8], [9]. Their lipophilic nature has also made cyclodextrins attractive as carrier molecules for hydrophobic drugs [10]. Given the increasing interest in cyclodextrins for human therapeutic purposes and the importance of cholesterol in peripheral auditory physiology, it is prudent to learn the effects that systemically administered cyclodextrins have on the inner ear. In the only published study examining HPβCD and hearing, threshold shifts were found in normal and NPC cats, but the nature of the underlying pathology was unexplored [11]. Additionally, the specificity of this effect to cats has yet to be determined, motivating work in other species to understand the iatrogenic potential of HPβCD. Here we investigate the ototoxicity of this drug in mice and describe hearing impairment with dramatic OHC loss after a single systemic dose.

## Methods

### Ethics Statement

Procedures involving the use of animals have been approved by the University Committee on Use and Care of Animals of the University of Michigan (protocol #08824).

### Animals and Treatments

Inbred FVB/NJ mice were chosen for this study because of their relatively good hearing with no particular sensitivity to age-related hearing loss [12]. Mice, ages 4–6 weeks, were injected with 2-hydroxypropyl-β-cyclodextrin (HPβCD; H107, Sigma-Aldrich) solution or vehicle (control) subcutaneously on the back. The HPβCD was diluted in sterile 0.9% NaCl and injected at a dose of 4,000 or 8,000 mg per kg body weight. These doses were chosen based on prior work showing therapeutic effects in NPC mice and hearing loss in NPC cats [8], [11], [13], [14]. Controls were given a volume of 0.9% NaCl equivalent to the volume of an 8,000 mg/kg HPβCD dose.

### Audiometry

Auditory brainstem response (ABR) and distortion product otoacoustic emission (DPOAE) tests were conducted essentially as described previously [15]. Mice were initially anesthetized with ketamine (65 mg/kg, IP), xylazine (3.5 mg/kg, IP), and acepromazine (2 mg/kg, IP), and additional ketamine and xylazine were given as needed to maintain depth of anesthesia. For each animal, DPOAE testing was performed immediately before ABR assessments [16]. Mice were maintained on a heating pad during recordings in an electrically and acoustically shielded booth. For DPOAE tests, a tube housing the end of a probe-tube microphone (ER 10B+, Etymotic Research, Inc.) connected to two speakers was fitted to the left ear. Two continuous acoustic tones (F1 and F2) were delivered simultaneously from the speakers while recording the microphone signal. This was accomplished using Tucker-Davis System 3 hardware. The frequency of F2 was 1.2 times that of F1, and the level of F2 was 10 dB below that of F1. The geometric mean of the tone-pair frequencies and the level of the F1 tone are used to describe the stimuli. Stimuli were presented at 8, 16, or 24 kHz at levels of 10–80 dB SPL (in 5 or 10 dB steps), and the amplitude of the 2F1–F2 distortion product was measured from the microphone signal. For ABR tests, needle electrodes were inserted in the skin at the vertex of the head and ventral to each ear. Tucker-Davis System 3 was used to deliver acoustic stimuli and to record electrode potentials. Test frequencies were 4, 16, and 32 kHz, which correspond to apical, middle, and basal thirds of the cochlea, respectively. Tone bursts 15 ms in duration with 1 ms rise/fall times were presented at a repetition rate of 10 per second. The stimuli were delivered via a speaker tube directed at the opening of the left ear canal. The level of the tone bursts was manipulated to find the lowest level (in dB SPL) that evoked a response and the level 5 dB below this failing to evoke a response. Threshold was determined to be between these two values. Up to 1024 responses were averaged for each stimulus level. Both systems were calibrated in a closed volume approximating that of a mouse ear canal, using a reference condenser microphone (Brüel & Kjær 1/8^th^ inch, Type 4138) and a lock-in amplifier (Stanford Research Systems SR830) over a broad frequency range spanning the reported results.

### Organ of Corti Whole Mount Preparations

Mice were anesthetized by intraperitoneal injection of pentobarbital and transcardialy perfused with PBS followed by 4% paraformaldehyde. Temporal bones were excised and decalcified. Preparations of the organ of Corti were treated with 0.3% Triton X-100 in PBS with 5% normal donkey serum and incubated with goat anti-prestin (1∶100, sc-22692, Santa Cruz Biotechnology, Inc.) overnight. The preparations were stained with AlexaFluor 488 donkey anti-goat secondary antibody (1∶500) and AlexaFluor 594-tagged phalloidin (1∶100) to label filamentous actin and imaged on a Zeiss LSM 510-META laser scanning confocal microscope. To quantify the extent of hair cell loss, successive 0.19 mm fields were evaluated on an epifluorescence microscope for the percentage of missing hair cells, determined from the presence or absence of phalloidin labeled hair bundles. For cholesterol staining, microdissected organs of Corti were incubated in a solution to quench autofluorescence (1.5 mg/ml glycine, 1% bovine serum albumin, and 0.02% saponin) followed by filipin (0.5 mg/ml, F-9765, Sigma-Aldrich); care was taken to perform staining procedures in dark. Freshly stained tissue was imaged under constant exposure conditions with images taken immediately after UV illumination to reduce bleaching.

### Plastic Sections

Temporal bones were excised, decalcified, dehydrated in a series of ethanol solutions, and embedded in JB-4 Plus (Electron Microscopy Sciences). The embedded tissue was sectioned at 3-µm thickness, and the sections were stained with a solution of Basic Fuchsin and Toluidine blue (both from Acros Organics) in 30% ethanol.

### Amplex Red Cholesterol Assay

Liver and cochleae were obtained from control and HPβCD-treated animals. Total lipids were extracted from 20 mg samples of liver (median lobe) and the membranous labyrinth of both cochleae from each animal. Tissue was diluted in 2∶1 chloroform:methanol (1 g:20 ml), homogenized using a Tissue Tearor (BioSpec Products, Inc.), and sonicated for 5 min. Four volumes of 0.9% NaCl were added and the samples centrifuged (2000 rpm, 10 min). The chloroform layer was removed from phase-separated samples and washed once with methanol/water/chloroform (94∶96∶4). The lower phase was vacuum dried, and the pellet was redissolved in 1X Amplex Red reaction buffer. Cholesterol level was determined using the Amplex Red Cholesterol Assay Kit (Invitrogen) according to manufacturer’s instructions.

## Results

### Acute Effects of Single Parenteral HPβCD Injection

**Figure 1:**
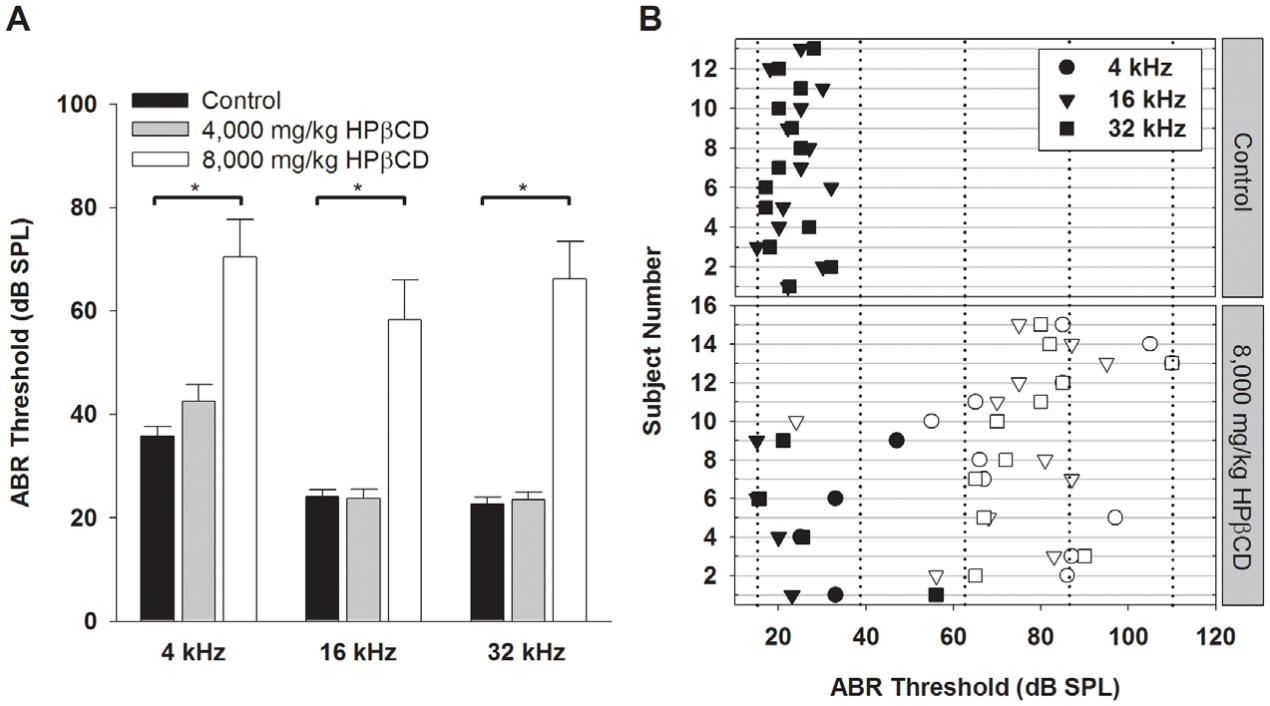
ABR thresholds are elevated one week following treatment with HPβCD. (A) Mean thresholds are shown for 4, 16, and 32 kHz. Control, N = 13; 4,000 mg/kg, N = 12; 8,000 mg/kg, N = 15. Error bars represent one standard error of the mean. * *P*<0.05. (B) Scattergram of thresholds shown by subject for control and 8,000 mg/kg treatment groups. Closed and open symbols in the drug treated group represent animals assigned to “unaffected” and “affected” categories, respectively.

**Figure 2:**
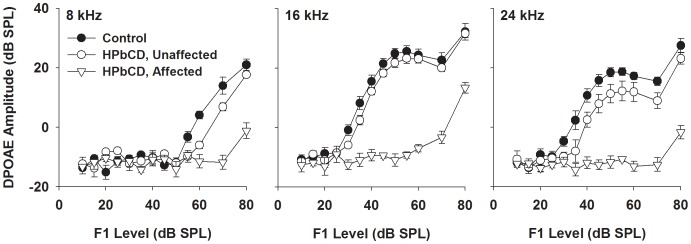
DPOAE responses are decreased one week after treatment with 8,000 mg/kg HPβCD. Treated animals were broken into two groups according to whether their ABR thresholds were affected by HPβCD. DPOAE amplitude is plotted against the F1 stimulus level for each of the three test frequency pairs with geometric means of 8, 16, and 24 kHz. Error bars represent one standard error of the mean.

To test the effect of HPβCD on peripheral auditory function, ABR thresholds and DPOAE amplitudes were measured one week after mice were given either the drug or the saline vehicle. For animals that received 8,000 mg/kg, mean ABR thresholds were elevated by about 35–45 dB at all three frequencies (Figure 1A). The individual ABR thresholds at this dose span a range from normal levels to profound impairment (Figure 1B). Interanimal variability may have reflected differences in pharmacokinetics (e.g. absorption, transport, permeation to the ear, and clearance of the drug) as well as slight procedural differences (e.g. placement of injection needle near fatty deposits or variable but small effusion out of injection site). Since the central goal of the study was to associate changes in hearing sensitivity with cochlear pathology, it was useful to take advantage of this range of effect by grouping the animals based on their deviation from control thresholds. This categorization was formalized using the 95% confidence interval associated with control responses. Animals with ABR thresholds outside the confidence interval for all three test frequencies were identified as “affected”. Using this criterion, 11 of 15 animals tested at the higher dose were categorized as affected (Figure 1B; open symbols). The remaining “unaffected” animals exhibited two or more thresholds within the control 95% confidence interval (Figure 1B; closed symbols). DPOAE tests on the high-dose animals bore out this finding. Mean DPOAE amplitudes were similar in control and unaffected animals across a range of stimulus intensities and test frequencies (Figure 2). In contrast, DPOAE levels of affected animals were dramatically reduced compared to controls. Thus, the audiometric findings point to a loss of OHC function in the affected animals. These effects were dose dependent. ABR thresholds for animals receiving 4,000 mg/kg HPβCD remained at control levels for all test frequencies (Figure 1A).

To determine whether deficits from the 8,000 mg/kg treatment were progressive, several animals were retested 2 weeks after injection. Four control and five treated animals (2 unaffected and 3 affected animals) were reexamined. There was no significant change in ABR thresholds (*P*>0.2). Average shift in ABR threshold across all test frequencies between the first and second week was 1.9+/−2.4 dB for control and −4.6+/−4.0 dB for HPβCD-treated animals. Similarly, DPOAE amplitudes were unchanged after a second week. Therefore, the damage caused by cyclodextrin was permanent and occurred within one week of injection.

**Figure 3:**
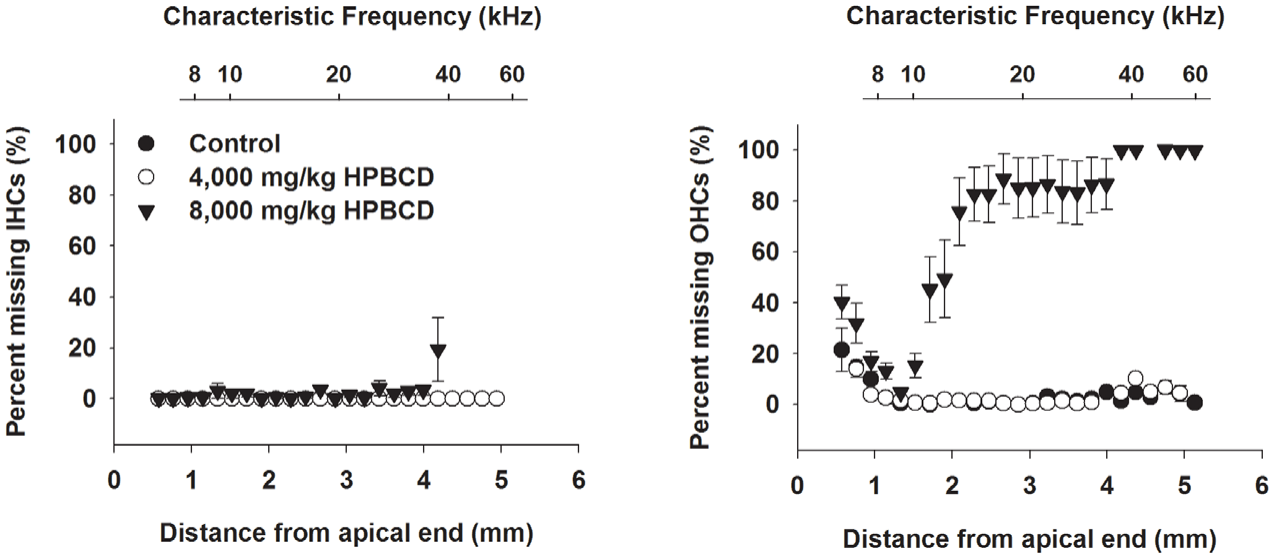
Cytocochleograms reveal substantial OHC loss one to two weeks after treatment with HPβCD. Scatter plots reflect averaged data from 4 to 10 animals per condition. Data from the 8,000 mg/kg treatment group were obtained from animals exhibiting elevated ABR thresholds (affected). Legend applies to both plots. Mapping of characteristic frequency to cochlear location is based on the formula *d* = 82.5*log(*f*)–56.5 [17], where *d* is the percent distance from the cochlear apex and *f* is characteristic frequency. The total length of the cochlea used for these calculations was 5.7 mm. The original mapping was derived from single-unit auditory nerve recordings with characteristic frequencies between 7.2 and 61.8 kHz, which correspond to locations between 10% and 90% from the apex. Accordingly, the frequency axis reflects only data from this range.

**Figure 4:**
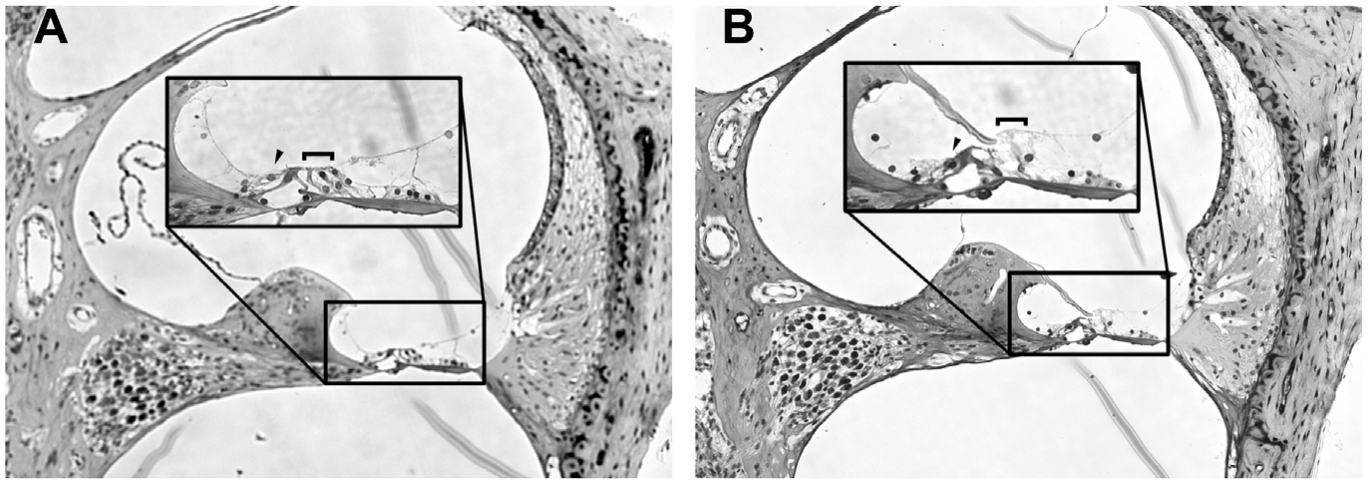
HPβCD-induced pathology appears restricted to the sensory epithelium. A mid-modiolar plastic section of the basal cochlear turn is shown by light microscopy for a control mouse (A) and an affected 8,000 mg/kg treated mouse (B). Insets show higher magnification view of the organ of Corti. Bracketed areas indicate the OHC region and arrowheads point to surviving IHCs. Images reflect similar results from three separate preparations in each treatment group.

To explore the contribution of inner ear damage to the observed ABR and DPOAE effects, histological analysis of the cochleae of control and HPβCD-affected mice was undertaken. The number of missing hair cells was counted to construct cytocochleograms of organ of Corti whole mounts (Figure 3). Animals given the 4,000 mg/kg dose exhibited hair cell counts that were indistinguishable from the controls, whereas those given 8,000 mg/kg displayed substantial hair cell loss. There was a modest amount of inner hair cell loss with the 8,000 mg/kg dose with the majority of the loss coming from a single animal. By comparison, there was a near complete loss of OHCs over the basal half of the organ of Corti. Widespread injury to basal OHCs can explain ABR and DPOAE shifts for frequencies 16 kHz and higher, but hair cell loss cannot easily explain elevated ABR thresholds at 4 kHz. In the mouse cochlea, the 4 kHz tonotopic location maps to the apical-most portion of the organ of Corti [17], and this region had a control-like compliment of hair cells in the 8,000 mg/kg HPβCD-treated animals. Plastic sections of cochleae from high-dose affected and control mice mirrored the cytocochleogram results with no other apparent defects (Figure 4). The histological findings show that OHC death primarily contributed to the loss of function at high frequencies, but the data also suggest more subtle effects of HPβCD on inner and/or outer hair cells in the apical organ of Corti, where hair cells were spared.

**Figure 5:**
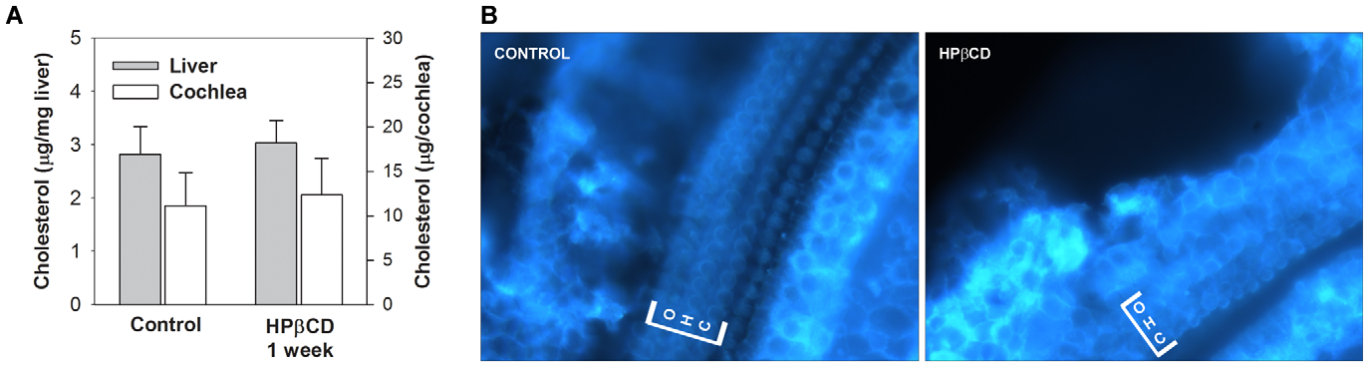
Cholesterol levels are unchanged one week after treatment with 8,000 mg/kg HPβCD. (A) Total cholesterol in liver and cochlea of control and HPβCD treated animals (N = 3 for each). Error bars represent one standard deviation from the mean. (B) Representative images of filipin stained organ of Corti. Mid-apical turn of control and HPβCD treated animals is shown.

Long-term effects of HPβCD on membrane cholesterol content could contribute to cell death and ongoing dysfunction in surviving cells. Although, parenterally delivered HPβCD is rapidly excreted [90% within 4 hours; 18], the pharmacokinetics of HPβCD uptake and removal from the cochlear duct remains unknown. Further, we know little about the biosynthesis and turnover rate of cholesterol in the cochlea, raising the possibility that a single dose could have long-term effects. We examined this possibility in two ways. Using a fluorometric assay, cholesterol levels in liver and whole cochleae were found to be the same in control and 8,000 mg/kg treated animals at one week (Figure 5A). Similarly, filipin stained organ of Corti revealed no difference in cholesterol level in surviving hair cells of the apical turn (Figure 5B). Consequently, chronic changes in cholesterol cannot easily explain the hearing deficits we observed. It appears that continuing chelation of cholesterol by HPβCD is not a factor and another direct or indirect effect of the cyclodextrin perturbs hair cell function.

Along these lines, HPβCD could have lasting effects on membrane proteins that are highly regulated by cholesterol. The OHC motor protein, prestin, for example, is exquisitely sensitive to both increases and decreases in membrane cholesterol content [7]. Prestin is a major component of the OHC lateral membrane. At a density of up to 8,000 particles per µm^2^ and a particle diameter of ∼8–10 nm, prestin contributes to approximately half of the membrane surface area [19]. Voltage-dependent conformation changes in prestin are believed to underlie OHC motility, cochlear amplification, and DPOAEs. Loss of prestin, and thus the cochlear amplifier, results in a reduction of about 50 dB in hearing sensitivity [20]. *In vitro*, methyl-β-cyclodextrin shifts the voltage range of prestin-associated motility [7], raising the possibility that HPβCD-related hearing loss at low frequencies could be related to prestin dysfunction in surviving OHCs. Following treatment with 8,000 mg/kg HPβCD, the typical uniform circumferential distribution of prestin (as seen in confocal imaging) was disrupted in remaining OHCs from the apical turn (Figure S1). Non-uniform staining patterns were readily identifiable in 5 of 7 preparations from HPβCD-treated mice. Between 11% and 46% of apical OHCs in those preparations revealed at least one puncta. Aligned circumferential intensity profiles showed that the prestin puncta were strikingly consistent in size (Figure S1 F). Given the average circumference of an OHC in this location (21.25±1.1 µm), the diameter of these clusters was estimated to be about 2 µm. The aberrant prestin pattern could itself be involved in the loss of OHC function and even OHC death [21], but it could also be a symptom of ongoing pathological processes induced by HPβCD.

### Chronic Effects of Weekly Parenteral HPβCD Injection

**Figure 6:**
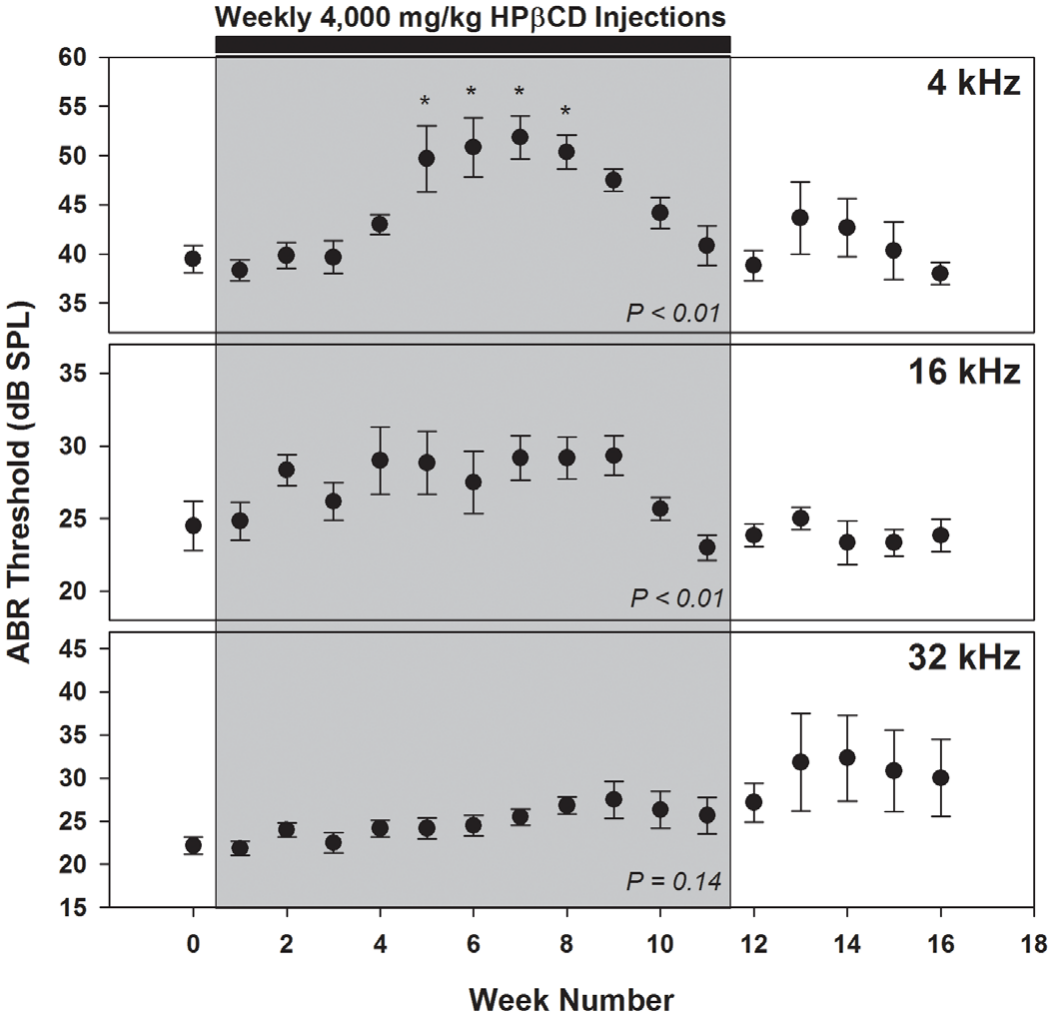
Weekly dosing with 4,000 mg/kg HPβCD results in a temporary elevation of low-frequency ABR thresholds. Thresholds for 4, 16, and 32 kHz stimuli were recorded weekly over a 16-week time period. Baseline responses were measured at Week 0. Injections began 5 days before the ABR measured on Week 1 and continued in this manner up to Week 12 (gray box). ABR measurements continued for an additional 4 weeks after cessation of the HPβCD injections. Error bars reflect one standard error of the mean (N = 6). Statistical significance was examined with one-way ANOVA (*P* values shown on plots). A Dunnett post-hoc test was used for pairwise comparisons between the baseline response on Week 0 and later time points (asterisk indicates *P*<0.05).

Low-dose HPβCD has no statistically significant effect on ABR thresholds following a single injection of the drug. However, treatment of Niemann-Pick Disease would require chronic dosing with HPβCD to maintain cholesterol homeostasis. Therefore, we examined whether repeated injection with 4,000 mg/kg HPβCD would render this dose harmful to hearing. We found a minor degree of hearing loss at 4 kHz that recovered even while injections continued (Figure 6). A smaller yet statistically significant effect was also seen at 16 kHz, whereas thresholds at 32 kHz were unchanged. A control animal injected with saline and tested in parallel showed stable thresholds throughout the 16 week period (not shown). The recovery seen at 4 kHz raised the possibility that homeostatic mechanisms in the ear were capable of counteracting the negative effects of HPβCD, leading us to hypothesize that cessation of drug treatment might cause elevated thresholds to return. To the contrary, there was no reliable difference in thresholds from Week 12 to 16 at any frequency.

## Discussion

Parenteral treatment with HPβCD produced variable, but often dramatic, hearing loss in mice. About one-quarter of the animals given a single 8,000 mg/kg dose had thresholds that were in the control range, while the remaining animals had mean thresholds about 50 dB above the control mean. Affected animals given 8,000 mg/kg lost most of their OHCs over the basal half of the cochlea, whereas the IHCs were left intact. The particular sensitivity of OHCs to HPβCD treatment could reflect their greater reliance on cholesterol or a greater sensitivity to perturbations in membrane composition.

While the role of lipids in cochlear physiology is largely unexplored, there is mounting evidence from a variety of *in vitro* studies that cholesterol influences the cochlear amplifier via effects on prestin, the OHC motor protein [7], [22]. The OHC lateral wall is an intricately arranged, multilayered structure consisting of the prestin-rich plasma membrane, a subsurface cistern, and a cortical lattice. Voltage-sensitive conformation changes in prestin are conveyed to the cytoskeletal lattice, generating the force necessary to alter cochlear mechanics and amplify low-level sounds. In the cochlea, prestin is specifically and highly expressed in the OHC lateral membrane; in contrast, expression in non-cochlear tissues is extremely limited [23]. The packing density of motor particles is extremely high, with estimates ranging from 2,500 to 8,000 particles/µm^2^ compared to theoretical limits of 10,000 to 15,000 particles/µm^2^ [19], [24], [25]. It is not surprising, then, that cholesterol concentration in the lateral membrane is low [7], [26] and that small perturbations in lipid levels could alter membrane integrity. The specificity of expression, dense packing in the OHC membrane, and sensitivity to cholesterol raise the intriguing possibility that a cholesterol-mediated effect of HPβCD on prestin is a key factor in the ototoxic effects we observed. Supporting this idea, knockout of prestin increases ABR thresholds by about 50 dB and eventually leads to hair cell death [21]. Alteration of prestin function could be involved in both the OHC death and the low-frequency hearing loss we observed, since prestin distribution was abnormal in surviving, apical OHCs (Figure S1). However, the pattern and timing of hair cell loss differs between prestin knockouts and HPβCD ototoxicity. In prestin knockouts, there is a progressive loss of OHCs beginning about four weeks after birth; by six weeks, the OHC loss approaches 60% in the basal third of the cochlea [21]. In these animals, about 30% of the IHCs also degenerate in the same basal region. By contrast, the HPβCD-related hair cell loss was specific to OHCs, occurred within one week, and encompassed more of the organ of Corti. Therefore, if prestin is involved in the death of hair cells due to HPβCD, then other contributing factors are probably at play. For instance, the membrane itself could be culpable. Mobilization of cholesterol could compromise the integrity of the membrane by removing a major lipid component. In addition, cholesterol has a major impact on ion channel function [27]. We have recently shown that cholesterol depletion inhibits voltage-gated potassium channel activity and enhances voltage-gated calcium channels in afferently innervated hair cells [6]. If these observations extend to OHCs, cholesterol depletion by HPβCD could alter ion homeostasis and lead to excitotoxicity. Channelopathies involving both IHC and OHCs preferentially affect OHC survival in some cases [28], raising the possibility that HPβCD-ototoxicity is related to excitability rather than membrane integrity.

Alternatively, HPβCD-induced ototoxicity may be related to mechanisms other than local depletion of cholesterol. Much of our understanding linking cholesterol to cochlear physiology comes from *in vitro* studies utilizing a methylated β-cyclodextrin rather than HPβCD. While β-cyclodextrins show some preference for binding cholesterol, HPβCD also mobilizes phospholipids and even proteins [29]. A lipidomic and proteomic analysis of the cochlea before and after HPβCD treatment will be essential to identifying targeted molecules and uncovering the mechanisms behind OHC death. It also will be important to consider that detrimental or beneficial compounds could be carried to or from the ear, since HPβCD was delivered parenterally. More work is needed to understand how HPβCD poisoned the cochlea in order to guide its therapeutic use or its use as a basic-science tool for experimentally damaging the cochlea.

For treatment of NPC, HPβCD will need to be delivered chronically. The previous study in cats [11] and our study in mice showed that the 4,000 mg/kg dose was innocuous to hearing when delivered once but led to a progressive shift in hearing thresholds when given repeatedly up to 7 weeks. The cat study stopped at this time point leading authors to conclude that chronic treatment with lower doses could be just as harmful as the higher single dose. However, we found that the cochlea had a remarkable ability to recover in the face of continued HPβCD dosing. The recovery precludes hair cell loss as a mechanistic explanation behind threshold shifts that peak around 7 weeks, since the mammalian cochlea is unable to spontaneously regenerate sensory hair cells. Even so, the same mechanisms could contribute to the permanent and temporary deficits, where minor insults from repeated low-doses are accommodated, but major disruption to the same processes from a single high dose results in permanent injury. Alternatively, the results could indicate at least two different HPβCD-sensitive processes, each with unique dose-dependent responses to the drug. In either case, the adaptive response offers a potential tool for monitoring the safety of HPβCD-treatment in clinical settings. For example, a clinical study using escalating repeated doses of HPβCD could monitor threshold shifts and recovery of hearing as an indicator for progression to the next higher dose in the regimen. Since the only other clinical pathology previously reported for these doses involves infiltration of foamy macrophages into the lung [18], routine measurement of hearing function could serve as a useful tool for noninvasively monitoring drug safety, if adaptive homeostatic processes in the ear were also found in other organ systems.

From a basic-science perspective, it is often useful to induce hair cell death in experimental animals. Currently, the only experimental protocols for eliminating mouse OHCs via systemic injection involve a multi-day drug course or the combination of an aminoglycoside and a diuretic. While easier to implement than a multi-day drug course, aminoglycoside/diuretic methods can cause significant mortality [30]. Even at very high doses, HPβCD is safe to vital organs [18], so it could be a more humane, simpler to use alternative. Further understanding of the pharmacokinetics and mechanism(s) of HPβCD ototoxicity will better enable us to employ this substance as a tool for experimental hair cell loss in research animals and to prevent hearing loss in humans.

## Supporting Information

Figure S1 **Aberrant prestin localization one week after treatment with 8,000 mg/kg HPβCD.** Confocal projections of control (A) and HPβCD-treated (B) organ of Corti. Mid-apical turns are shown stained with anti-prestin (green) and rhodamine-phalloidin (red). Circumferential profiles of prestin immunoreactivity in an exemplar OHC from a control animal (C’), a cell from an HPβCD-treated animal with a control-like appearance (C”), and a cell from an HPβCD-treated animal exhibiting a punctate staining pattern (D). Normalized intensity profiles are shown for control OHCs (E; N = 9) and HPβCD OHCs exhibiting one or more puncta (F; N = 14), where the peaks of the intensity profiles were aligned to the half-way point around the cell circumference.
(TIF)
