# Supplementary figures and images for: Hearing Loss and Hair Cell Death in Mice Given the Cholesterol-Chelating Agent Hydroxypropyl-β-Cyclodextrin
Source: PLoS One. 2012 Dec 28;7(12):e53280. doi: 10.1371/journal.pone.0053280 (PMC3532434; doi:10.1371/journal.pone.0053280)

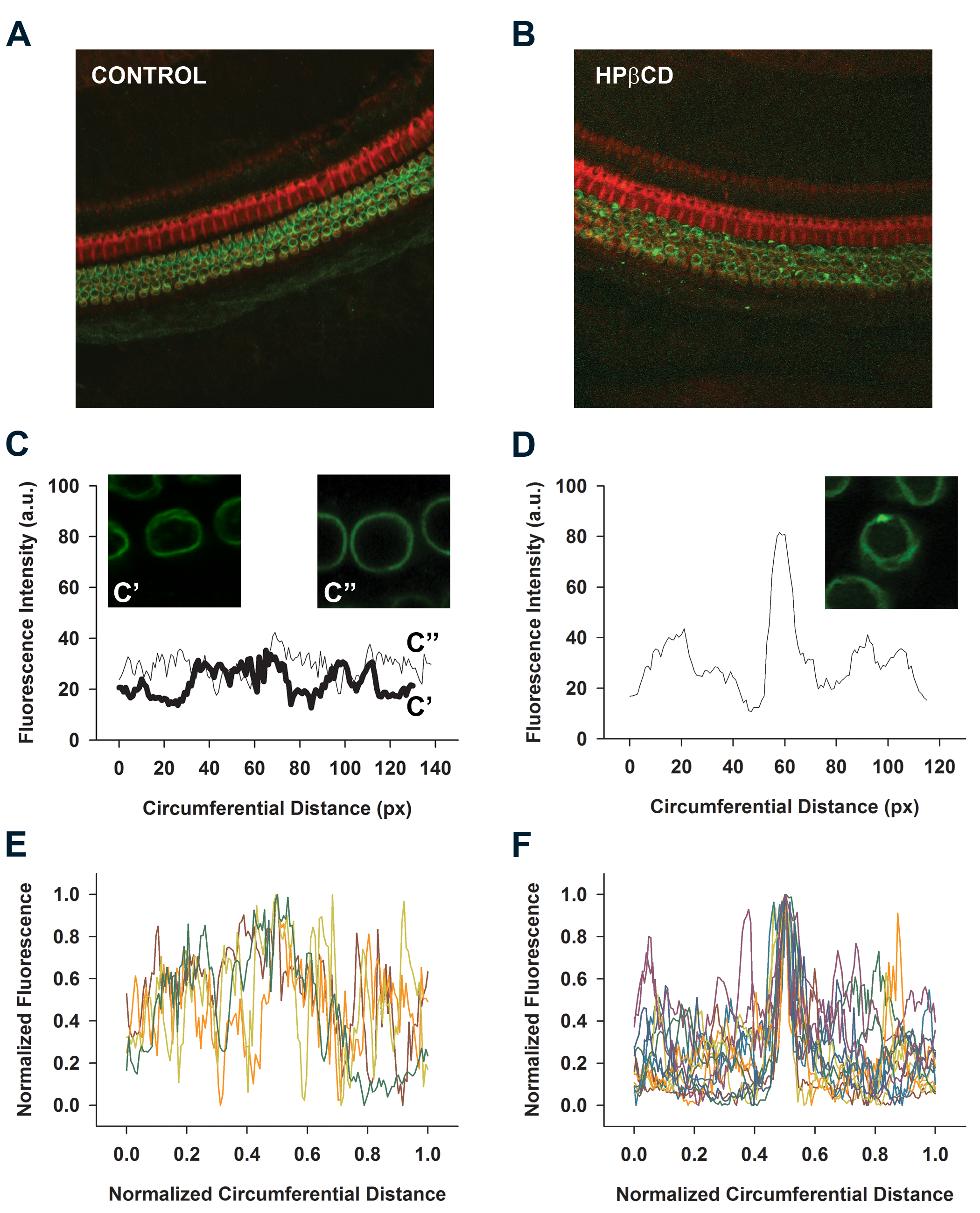

Supplement: Figure S1 — Aberrant prestin localization one week after treatment with 8,000 mg/kg HPβCD. Confocal projections of control (A) and HPβCD-treated (B) organ of Corti. Mid-apical turns are shown stained with anti-prestin (green) and rhodamine-phalloidin (red). Circumferential profiles of prestin immunoreactivity in an exemplar OHC from a control animal (C’), a cell from an HPβCD-treated animal with a control-like appearance (C”), and a cell from an HPβCD-treated animal exhibiting a punctate staining pattern (D). Normalized intensity profiles are shown for control OHCs (E; N = 9) and HPβCD OHCs exhibiting one or more puncta (F; N = 14), where the peaks of the intensity profiles were aligned to the half-way point around the cell circumference. (TIF) [file pone.0053280.s001.tif]
